# Haploinsufficiency for Steroidogenic Factor 1 Affects Maternal Behavior in Mice

**DOI:** 10.3389/fnbeh.2016.00131

**Published:** 2016-06-28

**Authors:** Tanja Spanic, Neza Grgurevic, Gregor Majdic

**Affiliations:** ^1^Veterinary Faculty, Institute for Preclinical Sciences, University of LjubljanaLjubljana, Slovenia; ^2^Institute of Physiology, Medical School, University of MariborMaribor, Slovenia

**Keywords:** mice, stress, steroidogenic factor 1, maternal behavior

## Abstract

Steroidogenic factor 1 (SF-1), officially designated NR5A1, is essential for gonadal and adrenal development and for the normal structure of the ventromedial hypothalamus (VMH), as demonstrated by SF-1 knockout mice (SF-1 KO), but much less is known about the possible effects of haploinsufficiency of the SF-1 gene. In the present study, maternal behavior in SF-1 KO heterozygous mice was evaluated. Behavioral tests revealed that SF-1 KO heterozygous females have impaired maternal behavior. In comparison to wild-type (WT) females, SF-1 KO heterozygous females retrieved significantly fewer pups into their nests, latency to retrieve and crouch over the pups was longer, and their nests were lower quality. As suggested by previous studies full dosage of SF-1 gene is needed for appropriate stress response and expression of brain-derived neurotrophic factor (BDNF) in the brain, and this might present a mechanism through which maternal behavior in SF-1 KO heterozygous females is impaired.

## Introduction

Species that give birth to altricial young, completely dependent on the parents’ care, protection and nutrition, develop strong parental behavior. In mammals, including humans and mice, both parents display parental behavior, although females usually have the primary role. In the conventional view, maternal behavior is stimulated by parturition and lactation accompanied by rapid changes in levels of sex hormones and brain neurotransmitters (Numan, 1999). Although hormones are believed to be the most important in preparing the brain for maternal behavior, a certain basic level of maternal responsiveness exist in virgin mice without previous experience with newborns. Moreover, adult ovariectomized females express maternal care in the absence of gonadal hormones, indicating a sufficient activation of the brain regions responsible for this behavior (Gandelman, 1973; Ehret and Schmid, 2009; Stolzenberg and Rissman, 2011; Kercmar et al., 2014). The medial preoptic area (MPOA) and bed nucleus of the stria terminalis (BST) are two brain nuclei reported as being activated during maternal behavior. These two brain regions project to different brain areas, most strongly to the lateral septum (LS), the medial hypothalamus (MH), and the mesotelencephalic dopamine system, and act together in switching between avoidance and attraction toward the pups (Lee et al., 1999; Sheehan et al., 2001; Kuroda et al., 2007). Interestingly, all these brain structures are also influenced by stress, and stressors could influence parental behavior. The stress response in mammals requires the normal function of hypothalamic-pituitary-adrenal (HPA) axis. Adrenal secretion is stimulated by the pituitary adrenocorticotropic hormone (ACTH), which is regulated by the hypothalamic corticotrophin-releasing hormone (CRH). Corticosterone, the main glucocorticoid in mice, acts on a broad range of target tissues including those of the brain (McEwen et al., 2015) and could also affect maternal behavior (Meek et al., 2001; Vilela and Giusti-Paiva, 2011; Pereira et al., 2015).

Steroidogenic factor 1 (SF-1), officially designated NR5A1, is essential for gonadal and adrenal development and for the normal structure of the ventromedial hypothalamus (VMH; Ingraham et al., 1994; Luo et al., 1994; Morohashi et al., 1994). The haploinsufficiency of SF-1 disrupts adrenal development and leads to impaired stress response. SF-1 knockout mice (SF-1 KO) heterozygous females have smaller ovaries and smaller adrenal glands with hypoplastic and disorganized medullae. Compensatory mechanisms such as cellular hypertrophy and increased expression of the rate-limiting steroidogenic protein StAR, help to maintain adrenal function at near-normal capacity under the basal conditions. However, under different stressful conditions, adrenal glands do not provide an adequate stress response and corticosterone levels could not reach levels of this hormone observed in stressed wild-type (WT) mice (Bland et al., 2000a,b, 2004).

Apart from stress response, there have been no previous studies observing the behavior of SF-1 KO heterozygous mice. Furthermore, in our laboratory, smaller litter sizes in SF-1 KO heterozygous females were observed, which led us to study the maternal behavior of these females. In the present study virgin, intact and inexperienced SF-1 KO heterozygous females and WT females were thus exposed to foster pups and observed in a standard maternal behavior test with the aim of determining whether haploinsufficiency for SF-1 impairs maternal behavior in mice.

## Materials and Methods

### Animals and Housing

Mice were housed at the Center for Animal Genomics, Veterinary Faculty, Ljubljana, under the standard conditions: 12:12 LD cycle with a temperature between 21 and 23°C and with food (Harlan Teklad Diet 2016, Harlan, Milan, Italy) and water *ad libitum*. The EU Directive 2010/63 and NIH guidelines were observed during all experiments, which were approved by the Veterinary Commission of Slovenia.

All tested females (SF-1 HET F *n* = 10; WT F *n* = 8) were housed in groups of 3–5 animals. All tested mice were ovary-intact and had no previous experience with mating, pregnancy, or pups. From postnatal day 67 (P67) their estrous cycle was followed via the collection of vaginal wet smears. On the first diestrus after P70, mice were tested for maternal behavior over two consecutive days. Previous studies in mice showed that maternal behavior in naïve females does not depend on different hormonal status and can be expressed even in the absence of hormones (Gandelman, 1973; Ehret and Schmid, 2009; Stolzenberg and Rissman, 2011; Kercmar et al., 2014); therefore, it is assumed that a different stage of the estrus cycle between the first and second tests did not significantly impact results.

### Genotyping

A piece of tissue from the ear (ear punch system was used for identification of animals) was digested in 200 μl PCR DNA buffer (Promega, Madison, WI, USA), with 0.15 mg Proteinase K (Sigma-Aldrich, Steinheim, Germany) at 55°C overnight. For PCR reaction, 3 μl of lysate was used, as described previously (Luo et al., 1994).

### Maternal Behavior Test

Maternal behavior testing was performed in the last hour of the light phase of the light/dark cycle. The testing animal was habituated to the cage with nesting material (Nestlets^®^) at least 3 h prior to the test. The test began with a placement of three stimuli pups (same strain, male pups, 1–3 days after birth) on the opposite site of the cage, furthest from the nesting material. Each female mouse was tested twice, in the first test for 20 min and in the second test for 15 min (the second test was shorter because the animals were already experienced with pups). During the tests, the following parameters were scored with the Stopwatch program (Center for Behavioral Neuroscience, Atlanta, GA, USA): latency to the first visit to the pups, latency to retrieve each pup into the nest, duration of grooming and number of times grooming was initiated (inside and outside the nest) and crouching over the pups (duration and number of initiations of this behavior). As non-maternal behaviors, nest building, nest quality, and climbing on the rack of the cage were scored. All tests were performed by the same observer, who was blinded with regard to the genotype during testing.

### Statistical Analyses

All statistical analyses were performed using NCSS software (NCSS statistical software, Kaysville, UT, USA). To test differences between genotypes, repeated measures analysis of variance (ANOVA) was performed with the genotype as a between factor variable and tests as within factor variable, followed by *post hoc* Fisher LSD testing. In addition to repeated measures ANOVA, all parameters were also statistically analyzed by balanced ANOVA for the first and second tests separately to determine any potential effect of the estrous cycle on behavior. The difference in the number of mice expressing maternal behavior was tested with a chi-square test. All differences were considered statistically significant at *p* < 0.05.

## Results

**Figure 1:**
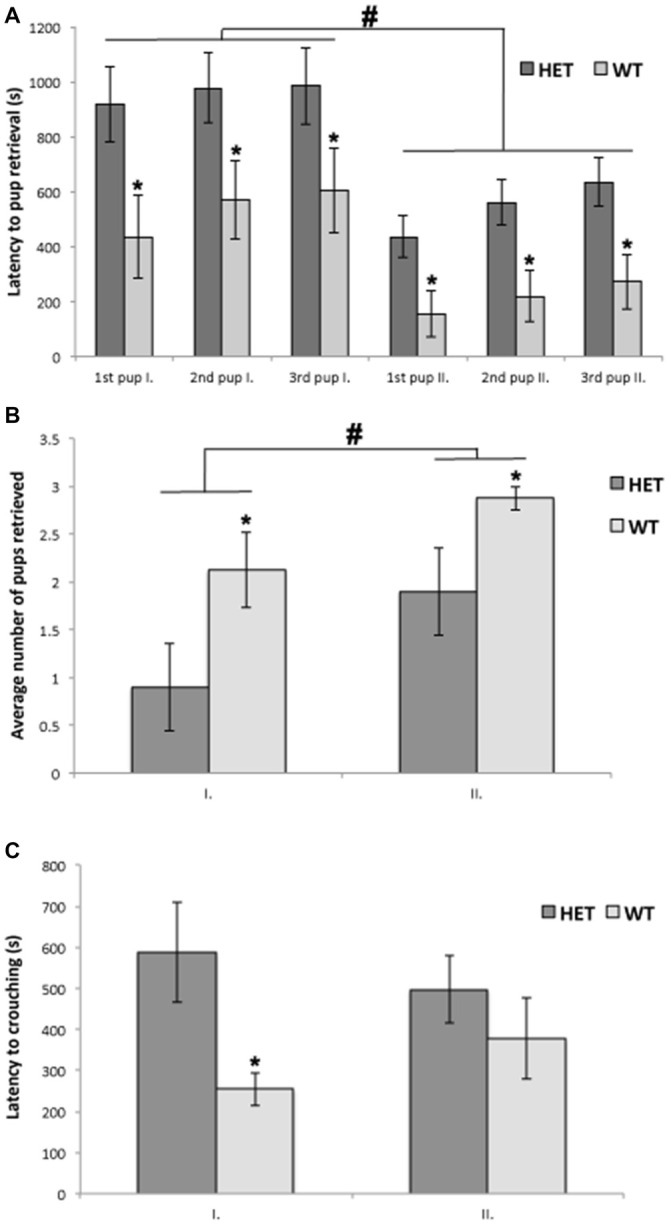
Wild-type (WT) mice (*n* = 10) retrieved significantly more pups into the nests (A; *p < 0.05) and had shorter latencies to retrieving pups into the nests (B; *p < 0.05) in comparison to steroidogenic factor 1 (SF-1) heterozygous females (*n* = 8) in both the first (I.) and the second tests (II.), although these behaviors did improve in second test in both genotypes (A,B; ^#^*p* < 0.05). Furthermore, latency to commence crouching behavior was significantly shorter in WT mice in comparison to SF-1 heterozygous mice (**C**; *p < 0.05), but only in the first test. All data are presented as Mean ± SEM.

All tested mice showed at least one of the pup-directed behaviors, such as grooming, crouching and retrieving, and none of the mice were aggressive toward the pups. All WT females (8 out of 8) were retrieving pups and 7 out of 10 SF-1 KO heterozygous females were retrieving at least one pup into the nest, although this difference was not significant in the chi-square test (*p* = 0.08). Repeated measures ANOVA revealed a statistically significant difference between genotypes (*p* < 0.05) in the number of retrieved pups and in the latency to retrieve pups into the nest. In both tests, WT females retrieved significantly more pups into the nest (*p* < 0.05) in comparison to SF-1 KO heterozygous females (Figures 1A,B).

Repeated measures ANOVA did not reveal a statistically significant difference between genotypes in duration, frequency, or latency for crouching behavior, although when crouching behavior was analyzed separately for the first and the second test, latency for crouching was statistically significantly different between genotypes. SF-1 KO heterozygous females were less maternal as they started to crouch over the pups significantly later (*p* < 0.05) than WT females, but only in the first test (Figure 1C).

**Figure 2:**
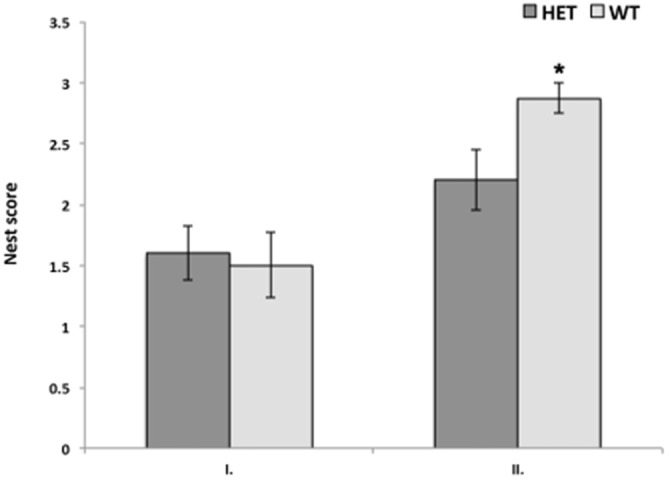
Nest quality was significantly better in WT female mice (*n* = 10) in comparison to SF-1 heterozygous mice (*n* = 8; *p < 0.05), although only in second test suggesting that quality of nest building improved with experience more in WT mice than in SF-1 heterozygous mice. All data are presented as Mean ± SEM.

In the quality of nest building, no significant difference was found with repeated measures ANOVA. However, if data were analyzed for the first and the second test separately, there was a significant difference between genotypes in the nest score only in the second test. Again, SF-1 KO heterozygous females built nests of significantly lower quality (*p* < 0.05) in comparison to WT females in the second test only (Figure 2).

## Discussion

Parental behavior is expressed in most mammalian species. Although it is well established that parental behavior is provoked by exposure to hormones during pregnancy in rats (reviewed in Rosenblatt et al., 1988) and sheep (reviewed in Dwyer, 2008), the development of the capacity to express parental behavior in mice is not yet fully understood (Kuroda et al., 2011). In the present study, the importance of the SF-1 gene dosage on some aspects of maternal behavior was examined. SF-1 heterozygous KO females retrieved significantly fewer pups into the nests; they were significantly slower in retrieving behavior and significantly slower in the initiation of crouching behavior in comparison to the WT females. Moreover, the quality of nests was also poorer in SF-1 KO heterozygous females, but only in experienced females in the second of the two tests. These data, therefore, suggests that SF-1 KO heterozygous females are poorer mothers than WT mice are.

There are very few studies examining the behavior of SF-1 KO heterozygous mice. Bland et al. (2000a,b, 2004) examined adrenal development and structure in SF-1 KO heterozygous mice and in one of these studies (Bland et al., 2000a) stress response was also studied, although only in male mice. The adrenal glands of SF-1 KO heterozygous mice were reportedly smaller and disorganized in these studies. Although the adrenal cortex had normal zonation, there was a marked cellular hypertrophy within the corticosterone-producing zone. In both sexes, most of the adrenal medulla was missing with only a few tyrosine hydroxylase-positive cromaffine cells being present. In female mice, those cells were located centrally and surrounded by the X zone, while in males, cromaffine cells were located eccentrically (Bland et al., 2000b). Consistent with altered adrenal structure, impaired response to different stressors was described. Acute stress (restrained for 30 min), chronic stress (food deprivation for 48 h) and inflammatory stress produced significantly lower plasma corticosterone levels in SF-1 KO heterozygous males in comparison to WT males (Bland et al., 2000a). Although these observations were reported for males only, it can be hypothesized that abnormal response to stress is also present in females since the adrenal glands in both sexes were markedly changed (Bland et al., 2000a,b, 2004).

Coping with stress is an important aspect of maternal behavior. In lactating rats, acute and chronic stress, induced by the treatment with dexamethasone, influences various maternal behaviors, such as increasing latency to build a new nest and retrieving the first pup into the nest, decreasing number of pups gathered into the nest, and decreasing crouching behavior (Vilela and Giusti-Paiva, 2011; Pereira et al., 2015). Therefore, blunted stress response due to hypoplastic adrenal glands in SF-1 KO heterozygous mice could underlie the impairment of maternal behavior, observed in the present study. It is possible that SF-1 KO heterozygous females did not cope properly with the stress that was caused by short social isolation before and during the test and exposure to pups as novel stimulus caused impaired stress responses that inhibited maternal behavior.

Within the brain, the SF-1 gene is specifically expressed only in the VMH (Ikeda et al., 1994). Studies with SF-1 KO revealed the importance of this gene during development since the complete loss of SF-1 gene results in the markedly altered cytoarchitecture of the VMH (Ikeda et al., 1995; Luo et al., 1995; Shinoda et al., 1995). Much less is known about the function of the SF-1 gene in the adult brain. Previous studies in adult SF-1 KO and in VMH-specific SF-1 KO showed that structural changes in the VMH could result in impaired social behaviors, such as anxiety, aggression, and female sexual behavior (Grgurevic et al., 2008, 2012; Zhao et al., 2008), in addition to impairment in energy balance regulation (Majdic et al., 2002). Although such structural changes as described in SF-1 KO homozygous mice are not present in SF-1 KO heterozygous mice (Shinoda et al., 1995; Dellovade et al., 2000; Tran et al., 2003), lower expression of brain-derived neurotrophic factor (BDNF) was observed in the VMH in heterozygous SF-1 KO mice (Tran et al., 2006). Interestingly, several studies described BDNF as a marker for changes in different social behaviors, such as depression and anxiety (Berton et al., 2006; Dalle Molle et al., 2012; Maynard et al., 2015; Mendez-David et al., 2015; Moser et al., 2015), which could also impact maternal behavior. Therefore, VMH function in SF-1 KO heterozygous mice could be affected by reduced BDNF expression in the VMH as reported previously (Tran et al., 2006) and this could potentially affect maternal behavior as observed in the present study. Interestingly, BDNF levels are not only dependent on the presence of the SF-1 in the VMH but are also influenced by stress hormones. Short-time immobilization stress (Rage et al., 2002) and the chronic restrain test (Naert et al., 2011) induced an increase in BDNF mRNA expression in the rat hypothalamus. Therefore, reduced BDNF levels in the brain of SF-1 KO heterozygous mice are not necessarily due to haploinsufficiency of SF-1, but could also result from an impaired stress response in SF-1 KO heterozygous mice. Regardless of the cause for low BDNF levels in SF-1 KO heterozygous mice, these changes could account for the impaired maternal behavior.

In conclusion, in the present study, the importance of the SF-1 gene in the regulation of maternal behavior is described. However, further studies will be needed to explore what the functions of the SF-1 gene in the adult brain are and to distinguish between the functions of the SF-1 gene during development and in adulthood.

## Author Contributions

TS and NG: performed experiments, contributed to analyses and writing of the manuscript. GM: designed the experiment, performed the analyses together with TS and NG: prepared a draft of the manuscript.

## Conflict of Interest Statement

The authors declare that the research was conducted in the absence of any commercial or financial relationships that could be construed as a potential conflict of interest.
